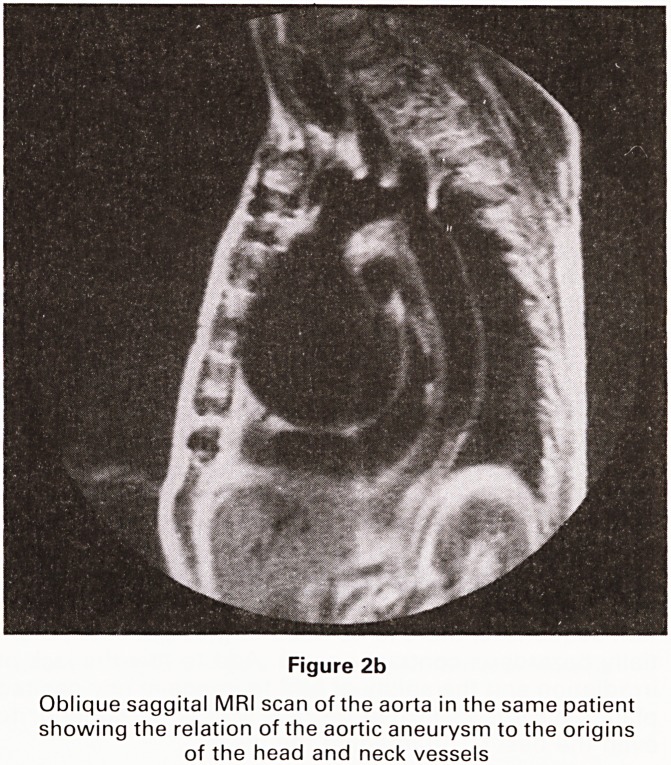# Cardiovascular MRI

**Published:** 1988-05

**Authors:** George Hartnell


Bristol Medico-Chirurgical Journal Volume 103 (ii) May 1988

### Cardiovascular MRI

**George Hartnell BSc, MB, ChB Bristol, MRCP, FRCR**

Of the many areas where the role of MRI has been investigated one of the most exciting, and potentially most important, is in the investigation of heart disease. Since the introduction of effective ECG gating devices, which allow image acquisition during a predetermined phase of the cardiac cycle, it has become possible to effectively "freeze" cardiac motion and to obtain very clear images of the structure of the heart. The development of new imaging sequences which show the flow of blood within the heart mean that the potential uses of MRI in the examination of the heart are almost limitless, at least in theory (Table 1). MRI has similar resolution to that of CT scanning but, because there is a large difference between the signal produced by heart tissue and flowing blood, which has no signal with most examination sequences, there is no need to give patients potentially hazardous contrast agents. Add to this the lack of irradiation and the ability of MRI to image in any desired plane and it is clear that Cardiac MRI is far superior to even the best CT in imaging the heart.

**Table 1**

**Current Indications for Cardiac MRI**

Aortic arch disease — Aneurysms
— Dissection
— Congenital
Ventricular function — Right and left ventricle
Ventricular aneurysms
Assessment of valve regurgitation
Atrial and ventricular septal defects
Cardiac masses — Tumours, thrombi.
Pericardial disease — Effusions, tumours, constriction.
Pulmonary hypertension/thromboembolic disease
Complex congenital heart disease
Bypass graft patency/flow rates

**Likely Future Indications for Cardiac MRI**

Coronary blood flow
Valve gradients
Coronary flow mapping (replacing angiography).

MRI can demonstrate all parts of the heart and is not affected by the presence of air around the heart. This is a great advantage over echocardiography, which although it has better resolution than MRI, is not satisfactory in a significant proportion of patients in whom a suitable acoustic window is not available. MRI can show almost any abnormality demonstrable by echocardiography, except possibly vegetations and calcified valves. MRI is superior to echocardiography in showing complex vascular abnormalities and some intracardiac masses. MRI is more accurate in assessing left ventricular function and much better at showing right ventricular function. Recently developed imaging methods allow MRI to demonstrate flow across valves and through congenital defects as clearly as the best colour flow Doppler equipment. The main drawback of MRI when compared to echocardiography is its great expense and the long time sometimes required to obtain images.

We have found that MRI is valuable in two particular situations. In the evaluation of patients with pericardial disease MRI has been superior to echocardiography and CT. It is the unique ability of MRI to freeze cardiac motion, to image in any plane and to visualise the entire heart, irrespective of any intervening bone or lung, that makes demonstration of the pericardium particularly clear (Figure 1). MRI reliably excludes significant pericardial thickening and can be used in the follow up of patients with pericardial constriction.

The assessment of thoracic aortic aneurysms has previously required contrast enhanced CT (usually requiring the administration of large volumes of contrast medium) and frequently also requiring angiography. We have found that the ability of MRI to visualise the aorta in any plane and to show the lumen without contrast media has in all of our cases provided as much information as CT and angiography combined (Figure 2a). In several cases MRI has provided extra information not shown by any other method, such as the full extent of dissections and the relationship of the carotid arteries to aneurysms (Figure 2b).

**Figure 1**

Transverse MRI scan of heart showing irregular thickening of the pericradium over the anterior surface of the heart

Our initial experience with cardiovascular MRI has been very encouraging and we hope to extend its applications. Cardiovascular disease is the most important cause of premature death in this country and the potential use of MRI to detect disease earlier and more safely than has hitherto been possible is only limited by the availability of suitable equipment.

**REFERENCES**

1. DINSMORE, R. E. LIBERTHON, R. R. WISMER, G. L. et al. (1980) Magnetic resonance imaging of thoracic aortic aneurysms: Comparison with other imaging methods. *AJR* **146**, 309–314.
2. HIGGINS, C. B. (1986) Overview of MR of the heart—1986. *AJR* **146**, 907–918.
3. SECHTEM, U. TSCHOLAKOFF, D. HIGGINS, C. B. (1986) MRI of the abnormal pericardium. *AJR* **147**, 245–252.

**Figure 2a**

Transverse MRI scan of aortic root showing considerable dilatation of the ascending aorta with a dissection flap separating the true lumen (right of picture) from the false lumen

**Figure 2b**

Oblique saggital MRI scan of the aorta in the same patient showing the relation of the aortic aneurysm to the origins of the head and neck vessels

## Figures and Tables

**Figure 1 f1:**
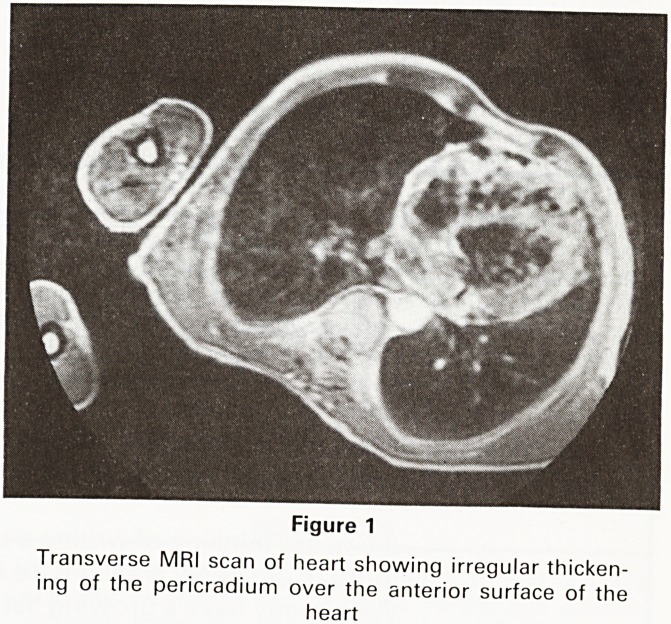


**Figure 2a f2:**
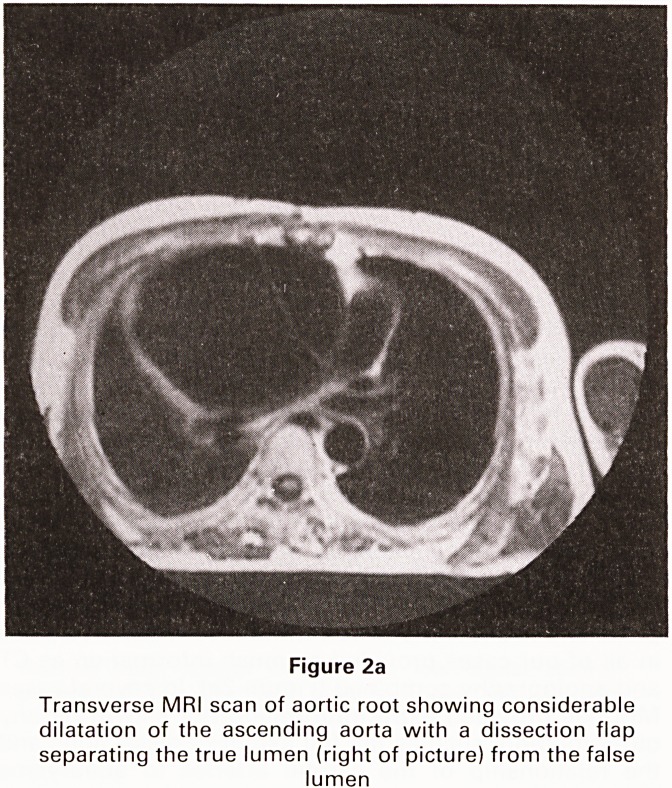


**Figure 2b f3:**